# Oral birch pollen immunotherapy with apples: Results of a phase II clinical pilot study

**DOI:** 10.1002/iid3.410

**Published:** 2021-02-23

**Authors:** Bettina Nothegger, Norbert Reider, Claudia E. Covaciu, Valentina Cova, Linda Ahammer, Reiner Eidelpes, Jana Unterhauser, Stefan Platzgummer, Elisabeth Raffeiner, Martin Tollinger, Thomas Letschka, Klaus Eisendle

**Affiliations:** ^1^ Department of Dermatology, Venereology and Allergology Medical University of Innsbruck Innsbruck Austria; ^2^ Department of Dermatology Venereology and Allergology Central Teaching Hospital Bolzano/Bozen Italy; ^3^ Department of Applied Genomics and Molecular Biology Laimburg Research Centre Ora Italy; ^4^ Institute of Organic Chemistry and Center for Molecular Biosciences Innsbruck University of Innsbruck Innsbruck Austria; ^5^ Clinical Chemistry and Microbiology Laboratory Tappeiner Hospital Meran Italy; ^6^ IMREST Interdisciplinary Medical Research Center South Tyrol Claudiana College of Health‐Care Professions Bolzano/Bozen Italy

**Keywords:** human, allergy

## Abstract

**Background:**

Seventy percent of patients suffering from birch pollen allergy (BPA) develop a pollen‐related food allergy (prFA), especially to apples, due to a clinically relevant cross‐reactivity between the major allergen in birch Bet v 1 and Mal d 1 in apples. Therefore allergen‐specific immunotherapy with fresh apples (AITA) could be a promising natural treatment of both BPA and prFA.

**Objective:**

To assess the clinical efficacy of immunotherapy by daily apple consumption for patients with BPA and prFA.

**Methods:**

A daily defined increasing amount of selected cultivars (Red Moon®, Pink Lady®, Topaz, Golden Delicious) was continuously consumed by 16 patients (12 female; median age; 50; range, 23–68 years), leading to increased intake of allergen over a period of at least 8 months. Specific IgE and IgG_4_ to Bet v 1 and Mal d 1, conjunctival and oral provocation tests, skin reactivity, and the average daily rhinoconjunctivitis combined symptom and medication score (CSMS) were measured during the peak birch pollen season.

**Results:**

After 8 months of therapy, patients showed increased tolerance to apples (*p* < .001) and a decreased skin reactivity to apples. Oral allergy syndrome to other birch prFA than apple also decreased (*p* < .05). Moreover, daily rhinoconjunctivitis CSMS declined by 34% (*p* < .001), as did conjunctival reactivity to birch pollen extract by 27% (*p* < .01), while specific IgG_4_ to Mal d 1 and Bet v 1 increased (*p* < .01).

## Graphical abstract

A 70% of patients suffering from birch pollen allergy (BPA) develop a pollen‐related food allergy (prFA), especially to apples. This is due to a strong clinically relevant homology between the major allergen in birch Bet v 1 and Mal d 1 in apples. Therefore a pilot trial using fresh apples for allergen‐specific immunotherapy to birch pollen was performed. After 8 months of therapy, patients showed increased tolerance to apples and other birch pollen‐related food allergens. Moreover, daily rhinoconjunctivitis combined symptom and medication score (CSMS) declined by 34% (*p* < .001), as did conjunctival reactivity to birch pollen extract by 27% (*p* < .01), while specific IgG4 to Mal d 1 and Bet v 1 increased (*p* < .01). In this small pilot trial daily apple consumption improved both birch pollen and related food allergies. If confirmed by a larger controlled trial apples could provide a natural, healthy and cost‐saving causal treatment for BPA.

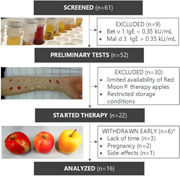

## 1 INTRODUCTION

Prevalence of birch pollen allergy (BPA) increased in the past decades^1^ leading also to a rise in cross‐allergies to raw plant foods containing PR‐10 proteins with homology to the major birch pollen allergen Bet v 1, known as birch pollen‐related food allergy (prFA).^2^ Especially Mal d 1 in apples has a strong clinically relevant cross‐reactivity to Bet v1,^3^ and induces oral allergy syndrome (OAS) in more than 70% of the patients with BPA.^4^, ^5^ This cross‐reactivity provides an opportunity to use Mal d 1 proteins in apples to potentially cure both BPA and birch prFA.^6^, ^7^

Apples are one of the most popular fruits in Europe, available throughout the year, with an annual consumption of 13.4 kg per person.^8^ Therefore, it is not surprising to frequently find more than 10 different cultivars at the supermarket showing different properties in color, size, taste, and allergen content. Importantly, apples are also associated with beneficial effects for human health.^9^, ^10^ Patients with prFA have to avoid fresh apples which is, until now, the only way of treatment, and further adversely affects their quality of life. To date, only a couple of apple cultivars are scientifically described to be tolerated by patients with mild prFA to apples (e.g., Santana, Elise).^11^, ^12^ A key treatment issue would be to find a way for patients to develop permanent tolerance to apples and simultaneously to birch‐pollen.

Specific immunotherapy of BPA with birch pollen extract has been successfully performed with licensed products for more than 100 years. Unfortunately, this hardly ever showed any effect on the concomitant prFA.^1^, ^13^, ^14^ Only recently, transient tolerance to apples after oral immunotherapy with apples was shown.^15^ Further, a sublingual immunotherapy study with recombinant Mal d 1 provided evidence for downregulation of specific Th2 response to both Mal d 1 and Bet v 1 and suppression of Bet v 1‐specific, cross‐reactive T cells.^16^ Another study showed a reduction of Bet v 1 specific serum IgE levels after oral exposure to Mal d 1 and induction of immune responses indicative for peripheral tolerance development.^4^, ^6^, ^17^, ^18^ Effects of long term apple consumption on BPA have not been studied.

A key treatment issue would be to find a way for patients to develop permanent tolerance to apples and simultaneously to birch‐pollen. Therefore built on these results, we developed a protocol based on continuously increasing apple consumption starting from small amounts of a low allergenic cultivar and finally reaching high amounts of highly allergenic apples.^6^ The objective of this present pilot study was to determine whether BPA and prFA can be reduced by continuous daily intake of fresh apples according to a previously published protocol. If feasible, immunotherapy with fresh apples (AITA) would provide a healthy, cost‐saving, and convenient way for BPA treatment.

## 2 METHODS

### 2.1 Study design

**Figure 1:**
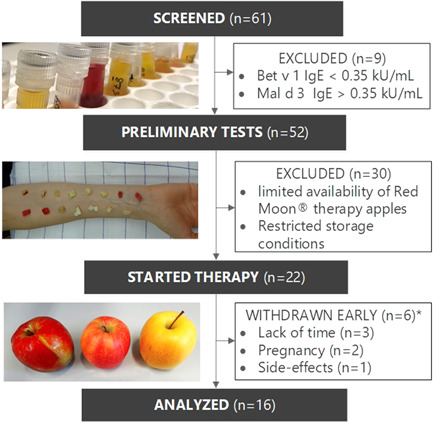
Clinical study design. Flow diagram showing the number of patients screened, treated, and analyzed in the study; *timepoint of withdrawal is shown in Figure 2

A detailed description of the concept is provided in a previously published manuscript.^6^ Briefly, 61 patients from the phase I trial who reported OAS after consumption of fresh apples were assessed for the present investigation (Figure 1). During screening, which consisted of serologic antibody assays, a standard skin prick test (SPT) and evaluation of the medical history, nine patients were excluded due to insufficient inclusion criteria (specific IgE to Mal d 3 >0.35 kU/ml or Bet v 1 <0.35 KU/ml). After that, skin reactivity to 23 apple cultivars was tested, followed by oral provocation tests (OPT) of selected apples, to identify suitable cultivars and dosages for therapy. Because of the limited availability of Red Moon® cultivars and restricted storage conditions, only the first 22 patients that underwent all preliminary tests for therapy could be selected for this trial. The study was performed after approval of ethics committees (Innsbruck and Bolzano, EK1116/2017) and written informed consent of all participants.

**Figure 2:**
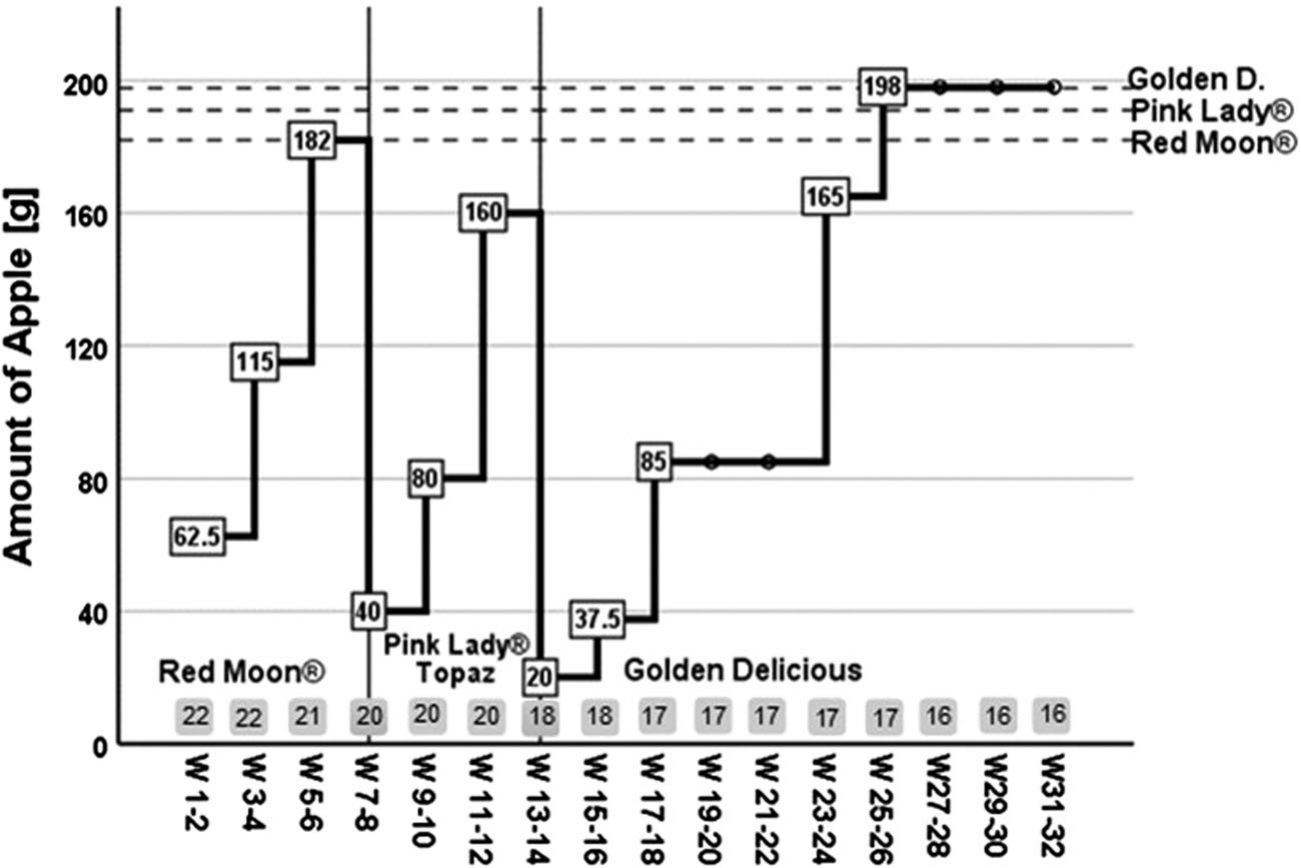
Immunotherapy over 32 weeks. Daily apple intake (amount in grams in the boxes) was continuously increased until an entire apple was tolerated, shown as dashed lines (Red Moon®, 182 g; Pink Lady®, 191 g; Golden Delicious, 198 g) or the apple sort was changed. The therapy started with the most acceptable dose of low allergenic Red Moon® over 6 weeks which was then replaced for another 6 weeks by Pink Lady® or Topaz, a moderate allergenic cultivar. After 12 weeks the cultivar was replaced by Golden Delicious for the remaining 20 weeks. The number of participants included is shown in the gray boxes

### 2.2 AITA with fresh apples

Based on the previous results, therapy was carried out as follows: AITA started in September 2018 with fresh harvested low allergenic Red Moon® cultivars. Patients began with the amount tolerated in the OPT. The daily apple portion was chewed carefully for 2 min, leaving some amount sublingually. Afterward, food and water intake had to be avoided for 15 min. If this was well tolerated, the amount of apple was continuously increased (maximum doubled) every 2 weeks until reaching one entire apple. After an average of 6 weeks, Red Moon® was replaced by moderate allergenic Pink Lady® apples for another 6 weeks, one patient receiving Topaz instead. Finally, the patients switched to high allergenic and well storable Golden Delicious apples, which were consumed for the last 5 months. Prior cultivar change, tolerability was again assessed by OPT to find the best suitable starting amount. Apples were handed out every 2 or 3 weeks and had to be stored between 2°C and 8°C until consumption. The median weight of one full fresh apple was analyzed for each cultivar by examining 200 freshly harvested apples (Red Moon® [182 g], Pink Lady® [191 g], Golden Delicious [198 g]). Adverse events were assessed through calls and after each apple delivery. Symptoms (e.g., itching or scratching mouth, itching nose, eye or ear and shortness of breath) were graded on a 4‐point scale (from 0, no symptoms, to 3, severe symptoms).

### 2.3 Serum antibody assays

Serum concentrations of total IgE, specific IgE, and IgG_4_ for Bet v 1 and Mal d 1 and IgE for Bet v 2 and Bet v 4 were quantified before and after AITA by ImmunoCAP (Phadia®250; Thermo Fisher Scientific) according to the manufacturer's specifications.

### 2.4 Skin prick tests

Twenty‐three different apple cultivars divided into red‐fleshed, old, and new cultivars named Bay 3484 Baya® Marisa (BM), Y103 Kissabel® (RF2), R201 Kissabel® (RF4), RS‐1 Red Moon® (RM), Luresweet Redlove® (RL), Goldparmäne (GP), Kanada Renette (KR), Tiroler Spitzlederer (TS), White Rosemary (WR), White Winter‐Calville (WW), Bonita (BO), Elstar Lb®87/1 (EL), Fuji Zhen® (FJ), Gala Buckeye®(GA), Gloster (GO), Golden Delicious Klon B (GD), Lb 17906 (LB), CIVG198 Modí® (MO), SQ159 Natyra® (NA), Pink Lady® Rosy Glow (PL), Santana (SA), Bay 4210 Sonnenglanz® (SG), Topaz (TO), and birch pollen extract (ALK‐Abelló) were skin tested before and after AIT according to Nothegger et al.^6^

### 2.5 Oral provocations

OPTs with Red Moon®, Pink Lady®, and Golden Delicious were performed within the AITA every time before a new apple cultivar was consumed and additionally, Golden Delicious was tested before and after AITA, according to Nothegger et al.^6^ To exclude unspecific and thus only temporary desensitization, OPT after AITA was exclusively performed after a strict avoidance of apples for at least 3 weeks after the endpoint of therapy. OPTs before and after therapy were performed in summer between June and August with stored apples. Apples were stored under standard commercial conservation conditions until use (cold storage at 2–8°C).

### 2.6 Conjunctival provocations

Conjunctival provocation tests (CPTs) with birch pollen extract (HAL‐Allergy Provo‐Test; HAL‐Allergy GmbH), diluted 1:10 with NaCl 0.9%, were performed outside the birch‐pollen season before and after AITA. Symptoms (itching, redness, tearing and foreign body sensation or chemosis) were recorded on a 4‐point scale (from 0, no symptoms, to 3, severe symptoms), according to Fauquert et al.^19^

### 2.7 Cross‐reactive plant foods

Cross‐reactivity to other plant foods was assessed before and after AITA by a questionnaire. Patients filled in a yes or no tolerability to the following 26 foods: almond, apricot, blackberry, blueberry, carrot, celery, cherry, coriander, dill, fennel, hazelnut, kiwi, melon, nectarine, parsley, parsnip, peach, pear, peanut, pepper‐green, plum, potato, raspberry, soy, strawberry, and walnut.

### 2.8 Pollen diary

The patients birch‐pollen diary in paper form was handed out for daily documentation of rhinoconjunctivitis and medication use (oral or ocular antihistamines, nasal corticosteroids) during the birch pollen season ranging from mid‐March to mid‐May. Daily symptom score and daily medication score were recorded according to the EAACI guidelines.^20^ Sympotms within the worst 3 consecutive weeks in April with the highest birch‐pollen load were compared, starting with the date of 3 consecutive days with a birch pollen count >30 PG/m^3^. Patients were scattered throughout the Tyrol and Southtyrol provinces, but pollen counts in the capital cities were higher in 2018 compared to 2019. For this reason, these results need to be interpreted with caution.

### 2.9 Statistics

All statistical calculations were performed using SPSS 26^21^ and GraphPad Prism 8 software (GraphPad Software).^22^ Demographic data and allergen‐specific immune responses (IgE, IgG_4_) are expressed as median, including ranges. SPT results are shown as median HEP‐Index diameter (allergen average wheal diameter divided by the positive control average diameter). Wilcoxon's matched‐pairs signed‐rank test was used to analyze differences before and after AITA. A *p* value of <.05 was considered significant.

## 3 RESULTS

### 3.1 Monitoring of AITA

Twenty‐two patients assessed for AITA had a median age of 48.5 years (range, 23–68), 68% were female. During AITA, six withdrew for the following reasons: lack of time (*n* = 3), pregnancy (*n* = 2), and one due to increased side effects. Patients started with an average amount of 62.5 g Red Moon® per day, as illustrated in Figure 2, whereby four patients were able to eat one full apple (182 g) without any complaints. After week 6, half to one entire apple was tolerated by 13/21 (67%) of the participants, 8/21 (24%) tolerated a quarter to half an apple. In week 7, Red Moon® was replaced by Pink Lady® except for one patient who ate Topaz instead, as Pink Lady® showed neither a reaction in SPT nor OPT. Patients started with an average daily amount of 40 g Pink Lady, the latter with 165 g Topaz. Three patients were already able to eat a three‐quarter piece of apple. At the end of week 12, 13/20 patients (65%) consumed three‐quarters to one full apple (191 g), and 7/20 (35%) tolerated less than a quarter of apple per day. After switching to the high allergenic cultivar Golden Delicious in week 13, patients initially tolerated a significantly lower amount (*p* < .001) compared with Red Moon®, eating a daily average of only 20 g apple. Golden Delicious was in general much worse tolerated than Pink Lady® or Red Moon®, especially within the first 4 weeks of AIT (weeks 13–16). All patients initially reported to have OAS but became slowly accustomed to the apple. Therefore, the ingested amount was only gradually increased so that 8/17 patients were able to consume a daily amount of three‐quarters to one full apple (198 g) after 10 weeks of therapy with Golden Delicious. In the 10 weeks that followed, daily intake was continuously increased, and symptoms started to decline. Only during the birch‐pollen season (week 28–31), some patients reported to less well tolerate apples. Interestingly, tolerance was better if the apple was eaten in the morning. At the end of AITA in week W32, 13/16 (81%) were able to eat three‐quarter to one full apple without problems, and the remaining 3/16 (19%) tolerated a quarter piece.

**Figure 3:**
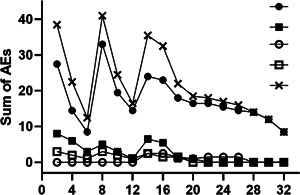
Monitoring of adverse events during allergen immunotherapy with apples. Adverse events 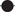 itching or scratching of mouth and throat (IS); 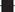 INEE; 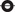 DSST; 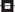 SB; 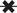 total symptoms were recorded in 2‐week intervals during AIT (TS), all rated as a sum on a scale of 0–3 (0: no to 3: severe symptoms). DSST, difficulty swallowing and swelling throat; INEE, itching nose, eyes or ears; SB, shortness breath

Most adverse events like discomfort, especially itching or scratching in the mouth and throat, occurred at the beginning of AITA with Red Moon® or after an apple was replaced, as can be seen from Figure 3. Itching nose, eyes, or ear and further shortness of breath or difficulty swallowing occurred less often and only mild. After the daily intake was increased or doubled, patients reported increased OAS in the first 3 days only.

#### 3.1.1 Assessment of AITA on birch prFA

**Figure 4:**
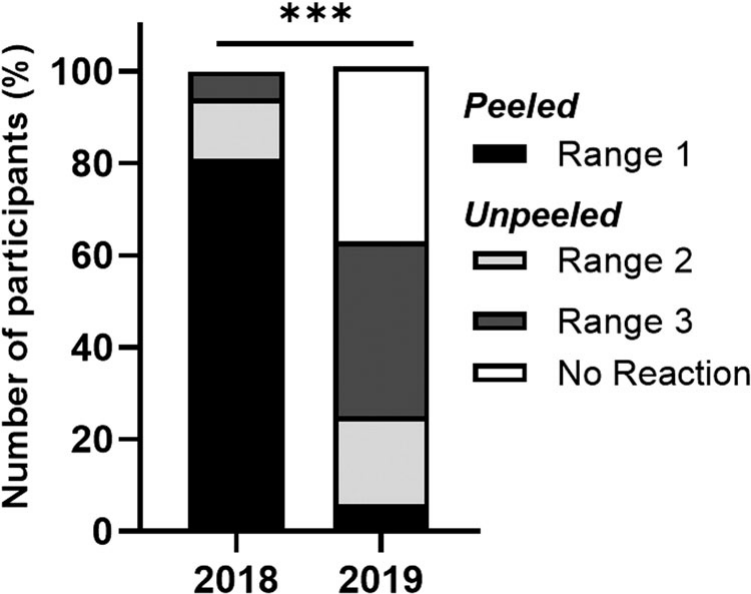
Number of participants (%) reacting to Golden Delicious before and after AIT. Patients (*n* = 16) were provoked with the high allergenic Golden Delicious before and after therapy in 2018 and 2019 for AIT assessment. The respective apple amounts that induced first OAS symptoms (e.g., itching/scratching) are divided into three ranges: range 1 (0.1–8.6 g) peeled, range 2 (13.6–43.6 g), and range 3 (83.6–163.6 g) unpeeled. In some cases, more than one full apple (163.6 + 100 g) was tolerated without any symptoms (=no reaction); ***p < .001, Wilcoxon signed‐rank test

After completion of AITA, patients were asked not to eat apples for at least 3 weeks, to avoid an unspecific desensitization effect. After that, follow‐up examinations started. OPT with Golden Delicious showed an increased significant tolerance of apples (*p* < .001) shown in Figure 4. Before therapy, 3/16 (81%) patients reacted to small, peeled quantities of <10 g apple. Afterward, 9/16 patients displayed first slight OAS after an amount of a quarter‐to three‐quarter piece of apple and six patients did not react at all. Only one patient again responded to less than 10 g of Golden Delicious.

SPT after AITA also showed a significant decrease in wheal size overall 23 apples tested (median HEP before 0.62 and after 0.58, −6%; *p* = .039), for all the therapeutic apples (median HEP before 0.65 and after 0.57, −12%, *p* = .021) and especially in Golden Delicious (median HEP before 0.83 and after 0.63, −24%, *p* = .008). SPT with birch extract declined by 12% (median HEP before 1.46 and after 1.28, *p* = .878).

#### 3.1.2 Effects on BPA

**Figure 5:**
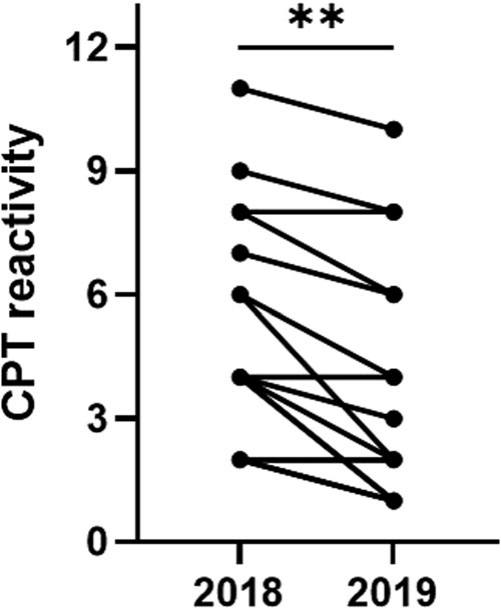
CPT reactivity. A CPT with birch pollen extract was performed before and after therapy. Shown is the sum of total symptoms (itching, redness, tearing and foreign body sensation or chemosis) of each patient (*n* = 16) in 2018 and paired for 2019; **p < .01, Wilcoxon signed‐rank test. CPT, conjunctival provocation test

CPT after AITA illustrated in Figure 5, displayed 27% significant reduction of conjunctival symptoms (mean CPT‐reactivity before 5.2 and after 3.8, *p* = .002).

**Figure 6:**
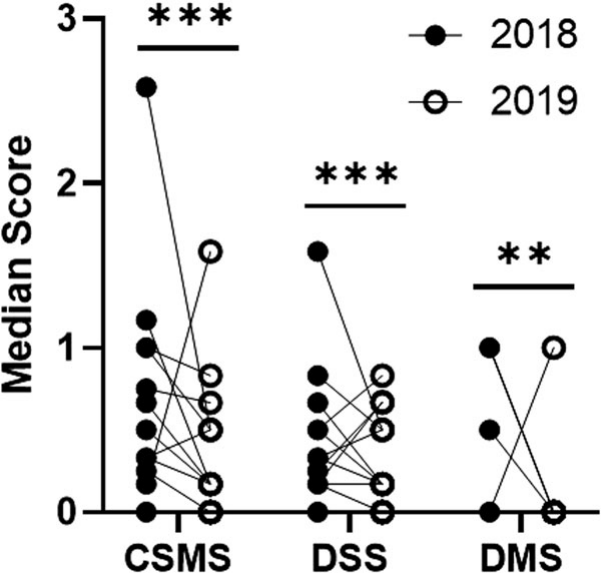
Allergy symptoms during birch pollen season. Comparison of the median daily CSMS, DSS, and DMS during the peak pollen period before and after therapy (*n* = 16) in 2018 and 2019; ***p < .001, **p ≤ .01, Wilcoxon signed‐rank test. CSMS, combined symptom and medication score; DMS, daily medication score; DSS, daily symptom score

Moreover, the mean daily combined symptom and medication score (CSMS) during the peak birch‐pollen season in April significantly declined by 34% (mean CSMS before 0.71 and after 0.47 *p* < .001) shown in Figure 6.

**Figure 7:**
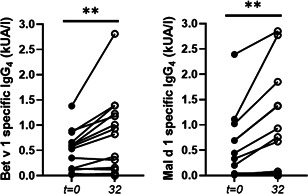
Bet v 1 and Mal d 1 specific IgG_4_ levels during allergen‐specific immunotherapy with apples. Sera were tested for allergen‐specific antibody levels before (*t* = 0) and after 32 weeks (*t* = 32) of therapy by ImmunoCAP analysis, **p = .01, Wilcoxon signed‐rank test

ImmunoCAP results after AITA are illustrated in Figure 7. IgG_4_ specific for Bet v 1 increased significantly from median 0.44 kUA/l (range, 0.02–1.38 kUA/l) to 0.60 kUA/l (range, 0.02–2.81 kUA/l), *p* = .013 as did IgG_4_ specific for Mal d 1 from median 0.12 kUA/l (range, 0.00–2.39 kUA/l) to 0.38 kUA/l (range, 0.00–2.85 kUA/l), *p* = .013). Specific IgE for both Bet v 1 and Mal d 1 did not change significantly.

#### 3.1.3 Evaluation of other Birch‐prFAs

Before entering the study, most patients (15/16, 96%) also showed OAS to other fruits, vegetables or nuts. Over 60% displayed OAS after eating cherries (11/16) or raw carrots (10/16), 6/16 (38%) reacted to apricot, hazelnut, kiwi, nectarine, peach, pear, or walnut, <30% to almond, celery, melon, peanut, plum, soybean, or strawberry (1–4/16).

After therapy, also OAS to other cross‐reactive plant‐food seemed to decrease aside from the apple allergy (*p* < .01 Wilcoxon rank test). OAS to kiwi, melon and peanut seemed to disappear completely, OAS to cherries improved in 6/11 patients (55%), to carrots in 4/10 patients (40%), to apricot in 4/5 (80%), to hazelnut in 3/5 (60%, to peach in 2/6 (33%), to pear in 4/6 (67%), and to plum in 3/4 (75%) of the patients. Tolerance to almonds, celery, nectarine, strawberry, soy, and walnut remained unchanged.

### 3.2 Compliance

Daily eating of apples was feasible, and all patients remained highly motivated. The daily consumption of the same apples over 8 months, however, was a challenge for a third (4/12) of the patients, they reported that they had to force themselves to eat the daily amount after switching to the high allergenic Golden Delicious. After 4 months, most patients (10/12) reported that they were tired of eating Golden Delicious every day and that they would change the apple cultivar if they could. Nevertheless, all patients were satisfied to eat apples experiencing hardly any or no symptoms with relish after years of not being able to eat apples. Indeed, after completion of the study patients continued to eat daily apples, but regularly changed within higher allergenic cultivars.

## 4 DISCUSSION

Based on previously published findings and following the protocol described,^6^ we performed an 8 months oral immunotherapy pilot trial with apples for the treatment of both BPA and prFA to apples. Our results seem to show the development of persistent tolerance to apples after daily and continuously increased intake and simultaneously an improvement of birch‐pollen induced rhinoconjunctivitis. Moreover, also OAS to other plant‐food cross‐reactive with birch pollen seemed to decrease.

Up to date, birch‐AIT has shown to be largely ineffective in treating prFA.^13^, ^14^ In contrast, oral immunotherapy with fresh apples achieved transient tolerance and SLIT with rMal d 1 was also effective for the treatment of birch prFA to apples.^14^, ^15^, ^17^ This study indicates for the first time that continuous consumption of apples by BPA patients with prFA to apples could both improve prFA and simultaneously may reduce birch pollen‐induced rhinoconjunctivitis.

After 8 months' daily consumption of a defined increasing amount of apple with increasing allergen content, 7/16 (52%) of the patients were able to eat on average 138 g more fresh apple, and a further 6/16 (37.5%) displayed no complaints after eating one entire high allergenic Golden Delicious. Tolerance to other cross‐reactive plant foods (e.g., cherry, carrot, or plum) also seemed to increase.

Even more interesting is a possible decrease in birch‐pollen induced rhinoconjunctivitis along with a significant reduced CPT‐reactivity with birch‐pollen extract. Moreover, we found an induction of IgG_4_ specific for Bet v1 and Mal d 1, indicating a specific immunologic response by reduced release of mediators in mast cells and basophils.^23^

Apples have an average weight of 135–228 g^24^ and contain 0.8–33 µg Mal d 1 protein/g fresh weight.^25^, ^26^, ^27^, ^28^, ^29^, ^30^ This leads most likely to an average consumption of 1 mg Mal d 1 protein/100 g of a middle to high allergenic apple (i.e., apples with Mal d 1 content of more than 10 µg/g), which is about a half apple. In a recent study, Pfaar et al.^31^ used a daily amount 100 µg Bet v 1 birch pollen product in a successful sublingual immunotherapy trail. By apple consumption, the 10‐fold amount of Mal d 1 protein is consumed, and it seems that this is sufficient to induce tolerance to both Mal d 1 and Bet v 1.

AITA was also successful in seven other patients that could not be included in the study due to restricted availability of Red Moon® apples. Hence, these patients were followed outside the study protocol with individually tailored commercially available apple cultivars with increasing allergen content (low‐middle‐high: Falchs Gulderling‐Pink Lady®‐Gala; Boskoop‐Pink Lady®‐Gala/Golden Delicious; Santana‐Topaz‐Gala) and showed increased tolerance to apples and decreased BPA symptoms after a minimum of 8 months apple consumption. This indicates that AITA is not only restricted to the cultivars used in this protocol.

As with any immunotherapy, it is important to maintain the treatment response. Therefore, patients should continue AITA to achieve permanent and long‐term efficacy with regard to both symptoms of BPA and birch‐prFA. Hence, we advised our patients after reaching the maintenance phase to subsequently consume one entire high allergenic apple (e.g., Golden Delicious, Gala, or Natyra) at least twice or three times a week. Moreover, according to the EAACI guidelines^32^ we recommend an AIT period of a minimum of 3 years for long‐term efficiency.

Daily eating of apples was well tolerated and safe and patients remained highly motivated, however, would prefer a greater variety of apples in daily consumption. Therefore, a higher selection and interchange of higher allergenic apple cultivars should be made possible in future subsequent studies. It should be reminded, that patients sensitized against the heat‐stable lipid transfer Mal d 3 protein (>0.35 kU/L) with the potential to induce severe anaphylactic reactions were excluded from this study. The protein also cross‐reacts with homologues proteins in Rosaceae fruits (e.g., Pru p 3 in peach). Whereas the instable Mal d 1 protein causes almost mild symptoms, as the so‐called OAS, in contrast to Mal d 3 that passes the gastrointestinal tract and can provoke more severe anaphylactic reactions.

In summary, the AITA protocol used in this study was feasible and effective for birch‐pollen allergic patients with associated OAS to apples. Prior analysis of different apple cultivars' allergenic potential enables to slowly start and increase the daily allergenic amount with a low allergenic apple cultivar followed by subsequent updosing with moderate and high allergenic cultivars until reaching on average one entire high allergenic apple. In this way, a safe and gradual allergen adjustment with low side effects could be achieved. Side effects mainly occurred at the beginning of AITA after apple cultivar change or after increasing the ingested amount.^6^ Therefore, we suggest that the first dose and each change to a new cultivar should take place under medical supervision. Apart from that, daily fresh apple intake is performed comfortably at home and provides a natural, healthy, time‐saving, and convenient way for allergy treatment. The avoidance of multiple hospital visits would be an advantage compared to the classical subcutaneous immunotherapy, the exploitation of apples with further positive effects on health^9^, ^10^ for immunotherapy would be an advantage to the classical sublingual immunotherapy. Further, as the price for a single apple lies way below one Euro or Dollar,^33^ this would also be cost‐effective.

Nonetheless, this study has several limitations. First, it is a small phase 2 pilot study with only 16 patients, second, it is known that the placebo effect of clinical improvement in controlled allergy trials is around 30%, further pollen concentrations were lower in the 2nd year, so effects on the symptom score have to be interpreted with caution.

In conclusion, our pilot‐study demonstrated that oral immunotherapy with fresh apples was feasible and safe for the treatment of both BPA and birch prFA. If the effect can be confirmed by a larger controlled phase III trial, daily apple consumption could provide a natural, healthy, and cost‐saving causal treatment for BPA and prFA with high potential for clinical application.

## CONFLICT OF INTERESTS

The authors declare that there are no conflict of interests.

## AUTHOR CONTRIBUTIONS

*Conception of the study*: Klaus Eisendle. *Study design and patient selection*: Bettina Nothegger, Norbert Reider, Claudia E. Covaciu, Martin Tollinger, Thomas Letschka, and Klaus Eisendle. *Laboratory analysis*: Valentina Cova, Linda Ahammer, Reiner Eidelpes, Jana Unterhauser, Stefan Platzgummer, Elisabeth Raffeiner, and Martin Tollinger. *Acquisition of data and patient care*: Bettina Nothegger, Claudia E. Covaciu, Norbert Reider, Thomas Letschka, and Klaus Eisendle. *Statistical analysis*: Bettina Nothegger and Klaus Eisendle. *Writing of the manuscript*: Bettina Nothegger, Norbert Reider, Thomas Letschka, and Klaus Eisendle. All the authors have read and approved the manuscript.

## Supporting information

Supporting information.

## Data Availability

The data that support the findings of this study are available from the corresponding author upon reasonable request.
