# Supplementary material for: Oral birch pollen immunotherapy with apples: Results of a phase II clinical pilot study
Source: Immun Inflamm Dis. 2021 Feb 23;9(2):503–11. doi: 10.1002/iid3.410 (PMC8127540; doi:10.1002/iid3.410)
Supplement: Supplementary file 1 — Supporting information. [file IID3-9-503-s001.docx]

**Supplementary data**

**Abbreviations**

prFA-pollen-related food allergy

AIT-Allergen-specific immunotherapy

BPA – Birch pollen allergy

OAS-oral allergy syndrome

OPT-oral provocation test

CPT-conjunctival provocation test

AITA – Allergen-specific-immunotherapy with fresh apples

SPT-Skin Prick-Test
